# Advancing immune checkpoint inhibitor rechallenge: key insights into efficacy, safety, and personalized strategies in advanced solid tumors

**DOI:** 10.3389/fonc.2026.1766921

**Published:** 2026-03-04

**Authors:** Shifen Lu, Zhong Xie

**Affiliations:** Department of Oncology, Oncology Hospital, Affiliated Hospital of Guangdong Medical University, Zhanjiang, Guangdong, China

**Keywords:** advanced solid tumors, biomarkers, ICI rechallenge, immune checkpoint inhibitors, immune-related adverse events, prognostic factors, safety management, treatment strategies

## Abstract

**Introduction:**

Immune checkpoint inhibitors (ICIs) have turned out to be a potent treatment of advanced solid tumor, but the issue of therapy discontinuation under the influence of the resistance, or immune-related adverse events (irAEs) is still a significant challenge. ICI rechallenge, which is a reintroduction of immunotherapy after initial failure is a favorable alternative whose guidelines are not standardized.

**Methods:**

This narrative review was a literature synthesis of the existing evidence drawn from PubMed, Web of Science, Embase, and Cochrane Library as up to July 21, 2025. We assesed real-world studies, retrospective cohorts, and meta-analyses, which examined patient selection criteria, rechallenge strategies, efficacy results, and safety profile across different types of solid tumors.

**Findings:**

The predictors of successful rechallenge include persistent initial response (progression-free survival ≥6 months), prolonged treatment-free interval (≥6 months), excellent performance status (ECOG-PS ≤1), and complete irAE resolution (Grade ≤1). The outcome of an after toxicity rechallage is superior to after progression (median PFS: 5.1 vs. 2.9 months). There is a better response to a combination of anti-angiogenics, chemotherapy, or radiotherapy strategies. However, the recurrence rate of irAE is 20%-60% and severe initial toxicities can be a reason to discontinue the drug permanently.

**Discussion:**

ICI rechallenge benefits the right patients significantly. We propose a clinical decision model that might assist in integrating both biological and clinical variables to base individualized rechallenge, but the standard set of criteria and possibilities to validate biomarkers remains in urgent need.

## 1 Introduction

With this great public health challenge of the exponentially growing global cancer burden, the emergence of immunotherapy and especially the use of immune checkpoint inhibitors (ICIs) has significantly revolutionized the treatment of advanced solid tumor disease. The repertoire of ICIs targeting key immune checkpoints has continued to grow since the landmark approval of ipilimumab in 2011, with programmed death-1 (PD-1), programmed death-ligand-1 (PD-L1), and cytotoxic T-lymphocyte-associated antigen-4 (CTLA-4) being the immunomodulators that have been developed and used. When ICIs prevent the occurrence of these suppressive checkpoints, T-cells are no longer held in check and anti-tumor immunity is restored. It has resulted in an impressive increase in overall survival (OS) and progression-free survival (PFS) in patients who have different advanced solid tumor types, such as melanoma, non-small cell lung cancer, renal cell carcinoma, and esophageal cancer over traditional chemotherapy or targeted therapies. Moreover, an average of 5 percent-15 percent of the patients have obtained a long-term disease-free survival or a functional cure, which indeed has brought a new era of immunotherapy of cancer (1–3).

Irrespective of these transformative successes, there are two main challenges facing the clinical use of ICIs at a large scale. To begin with, a large percentage of patients display the primary resistance, which is the inability to respond to the initial ICI treatment, and incidence rates differ depending on the tumor type and the treatment regimen (4–6). Although combination therapies may still apply to some initial non-responders, the resistance is still a challenge (5, 7). Secondly, a significant proportion of first responders develop acquired resistance or suffer intolerable immune-related adverse events (irAEs), which causes the need to discontinue treatment (8–11). These irAEs may severely affect the quality of life of patients and their life may be at stake, requiring clinicians to carefully weigh the benefits of therapeutic interventions against the possible risks. Addressing these critical limitations, the reintroduction of immunotherapy after initial ICI treatment failure or discontinuation, termed “ICI rechallenge”, has rapidly emerged as a pivotal research area and an urgent clinical dilemma in oncology. ICI rechallenge involves the readministration of the same or a different ICI agent as a subsequent treatment strategy (12–15). Preliminary evidence suggests that in patients with low tumor burden, initial ICI treatment can establish specific anti-tumor immune memory T cells (16, 17). During the subsequent “treatment-free interval” (TFI), exhausted T cells within the tumor microenvironment may gradually recover function, and immunosuppressive factor expression may decrease, partially restoring the body’s anti-tumor immune response (17–20). Crucially, immune selective pressure can drive tumor clonal evolution, leading to the exposure of novel tumor-associated antigens (neoantigens) that could create a new “immune response window” for the immune system (21–23).

However, despite the burgeoning recognition of ICI rechallenge’s clinical utility and its inclusion in treatment guidelines for certain cancer types (e.g., non-small cell lung cancer, colorectal cancer, melanoma, and urothelial carcinoma), current recommendations remain notably limited (24–31). Specifically, there is a distinct absence of unified patient selection criteria, consensus on optimal rechallenge timing, appropriate drug selection strategies, and standardized combination therapy models. Moreover, a comprehensive system of reliable biomarkers for predicting both efficacy and safety risks remains incomplete. These are critical gaps that have a huge negative effect on the standardized clinical use of ICI rechallenge. Hence, the current review attempts to syntactically review and study the available literature to comprehensively investigate the most important variables that affect the rechallenge outcomes of ICI. We will fully consider the criteria of patient selection (depth of response with initial treatment, treatment-free period, patient baseline condition, and progression of the disease), rechallenge strategies (monotherapy, combined with chemotherapy, targeted therapy, local therapy or cross-mechanism change over), and multi-dimensional safety management. Finally, this review aims to equip clinicians with more clinically sound evidence-based advice when making ICI rechallenge decisions, as well as, to give future prospective studies promising opportunities to enhance the standardization and individualization of ICI rechallenge in advanced solid tumors.

## 2 Literature search and selection

**Figure 1:**
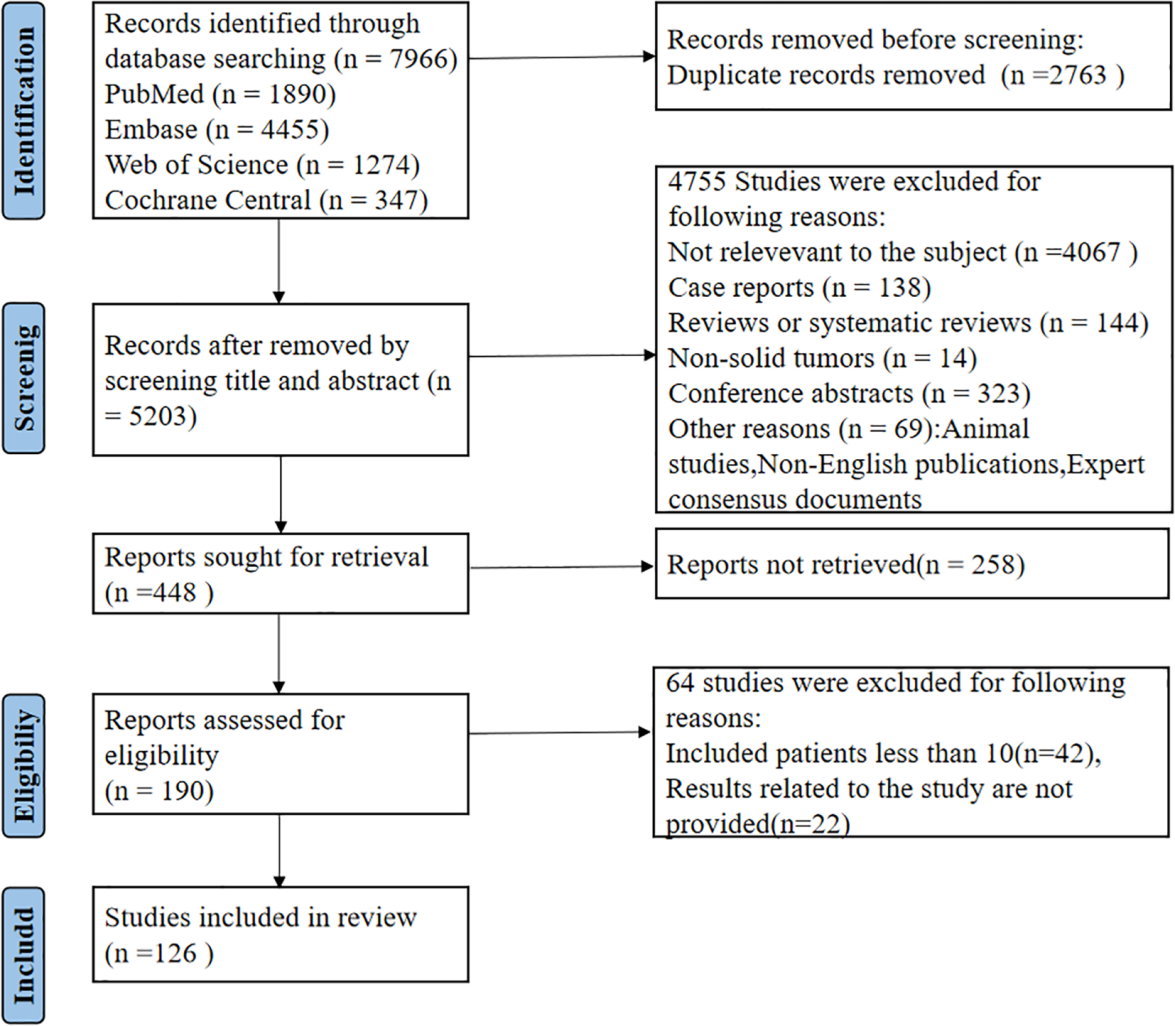
Study selection flowchart. This flowchart details the literature selection process for this systematic review. Initial records were identified through systematic database searches. After duplicate removal, screening of titles and abstracts excluded irrelevant studies. The remaining full-text articles were assessed for eligibility based on predefined inclusion and exclusion criteria, leading to the final set of studies included in the analysis.

In order to systematically and thoroughly assess the current situation in the research in relation to immune checkpoint inhibitor (ICI) rechallenge, the given systematic review followed a thorough literature search and selection procedure. Our intention was to determine all the high-quality clinical studies, real-world cohort studies, and meta-analyses to present a robust evidence base of ICI rechallenge in clinical practice and future research needs. To search, we used PubMed, Embase, Web of Science, and Cochrane Library, and the search cut-off date was July 21, 2025. In order to achieve a balance of breadth and accuracy, we carefully formed a search query, putting emphasis on the fundamental idea of ICIs, particular medication names (ipilimumab, nivolumab, pembrolizumab, atezolizumab, and durvalumab) and some of the phrases of rechallenge (e.g., rechallenge, retreading, readministration and repeated treatment). To fully capture the types of cancer, the search terms were broad terms of tumor and malignant tumor, and specific types of cancer such as non-small cell lung cancer (NSCLC), small cell lung cancer (SCLC), melanoma, renal cell carcinoma, hepatocellular carcinoma, gastric cancer and colorectal cancer. All these keywords were carefully put together with the help of Boolean operators (AND, OR) so as not to lose any relevant literature and to be highly specific. The careful study selection procedure that includes identifying records in the first stage, eliminating duplicates in the second stage, screening titles and abstracts in the third, and evaluating full-text on preestablished inclusion and exclusion criteria in the fourth step, is fully described in **Figure 1**. This eventually resulted in the end set of research used in this analysis. Such transparency and reproducibility of our search strategy is an essential aspect so that it can be verified by other researchers.

## 3 Prerequisites and patient selection for ICI rechallenge

Due to the potential of ICI rechallenge in select patient groups, accurately identifying those patients who are at the highest risk of rechallenge and whether and when to rechallenge them is an essential consideration in improving clinical efficacy. An extensive patient selection guide in ICI rechallenge is systematically offered in the form of overviewing main clinical factors, favorable factors, and their clinical implications and recommendations, which are listed in **Table 1**. This review critically examines the underlying requirements that affect ICI rechallenge outcomes such as the intensity of initial treatment response, TFI, cause of treatment discontinuation, patient performance status, disease progression characteristics, and history of irAEs to offer more refined patient selection criteria and decision-making advice to clinical practice.

**Table 1:** Key clinical factors influencing outcomes of ICI rechallenge.

Factor Category	Favorable Criteria	Clinical Implication & Recommendation	References
Reason for Initial ICI Discontinuation	Due to irAEs or clinical decision	Favorable efficacy & manageable safety: Rechallenge is recommended.	(32–36)
	Due to disease progression (PD)	Limited efficacy: Requires careful consideration; combination strategies are preferred.	(13, 37–39)
Depth of Initial Response (PFS_1_)	PFS_1_ ≥ 6 months	Strong positive predictor: High likelihood of successful rechallenge.	(40–45)
	PFS_1_ < 6 months (Primary refractory)	Poor efficacy: Routine rechallenge is generally not recommended.	(15, 37, 38)
Treatment-Free Interval (TFI)	TFI > 6 months	Superior outcomes: Allows immune reconstitution; rechallenge is favored.	(46–49)
	TFI ≤ 6 months	Reduced likelihood of benefit: Should be approached with caution.	(46, 47)
irAEs Status	Resolved to CTCAE Grade ≤1 & corticosteroids discontinued/tapered	Safety prerequisite for rechallenge: Can be considered.	(50–53)
	History of ≥Grade 3 myocarditis	High recurrence & mortality risk: Permanent discontinuation is advised (contraindication).	(54)
	Other unresolved ≥Grade 3 irAEs	High risk: Generally not recommended; requires individualized assessment.	(8, 52, 55, 56)
Performance Status (ECOG PS)	ECOG PS 0-1	Independent positive predictor: Greater potential for benefit and better tolerability.	(40, 49, 57–59)
	ECOG PS ≥ 2	Poor prognosis: High risk of disease progression, death, and poor tolerability.	(40, 58, 60)

### 3.1 Reasons for initial ICI discontinuation

The choice to continue or discontinue the initial ICI therapy is multifactorial, with three groups of situations, namely progressive disease (PD), irAEs, and clinical decisions (e.g., at the end of the planned treatment course). The risk-benefit profile of rechallenge varies in each situation, and it is essential to learn the cause of discontinuation to develop specific individual approaches. In most instances, patients whose primary therapy was discontinued on grounds of controllable irAEs or even because of deliberate clinical reasons tend to retain the potential of the immune system to be reactivated which implies they have a greater chance of responding to rechallenge although with a close attention to toxicity. Conversely, in patients with PD the usefulness of rechallenge should be less effective and more debatable, and more inventive approaches, such as cross-mechanism switching or combination treatment, may be pursued on a strict basis of patient selection to overcome deep-rooted resistance.

#### 3.1.1 Progressive disease

One of the most frequent causes of the initial discontinuation of the ICI treatment is that the malignancy has acquired or developed primary resistance mechanisms such that the tumor is now able to survive immune assault via various different pathways (13, 32, 33, 37). In a retrospective analysis of 144 patients with advanced NSCLC, Gobbini et al. reported that, compared to rechallenge after progression, rechallenge following discontinuation due to toxicity yielded significantly longer median progression-free survival (mPFS: 5.1 vs. 2.9 months) and overall survival (mOS: 2.1 vs. 1.0 years) (32). Furthermore, a meta-analysis by Xu et al., comprising 15 studies and 442 NSCLC patients undergoing ICI rechallenge, revealed that patients discontinuing initial ICI treatment due to PD had the lowest objective response rate (ORR), at only 11.4% (lower than 20% for irAEs-related discontinuation and 46.2% for PD occurring after initial ICI treatment completion) (13). A large-scale meta-analysis by Liu et al. (36 studies, 2026 patients) reported similar findings (33). For patients with PD, the risk of developing high-grade irAEs after rechallenge significantly increased by 4.97 times (OR, 4.97; 95%CI, 1.98-12.5, P<0.05), and the ORR was significantly reduced (OR, 0.48; 95%CI, 0.24-0.95, P < 0.05) (33). Patients whose immune systems were successfully activated and established durable immune memory during initial treatment (i.e., those with PD after initial treatment completion or irAEs-related discontinuation) may benefit more from rechallenge due to effective re-initiation of anti-tumor immune responses. Conversely, patients who experience PD during initial treatment may harbor primary or early acquired resistance mechanisms, leading to suboptimal rechallenge outcomes.

#### 3.1.2 Immune-related adverse events

irAEs represent another major cause of initial ICI treatment discontinuation. Unlike PD patients, those who discontinue due to irAEs typically have successfully activated immune systems with established anti-tumor immune memory. After complete resolution of irAEs, restarting ICI therapy holds promise for reactivating these immune responses, thereby conferring clinical benefits (32). In a systematic review (n=2026), patients who discontinued because of irAEs were found to be at much less risk of any-grade irAEs (OR, 0.01; 95%CI 0.00-0.25, P<0.05) and high-grade irAEs (OR, 0.19; 95%CI 0.15-0.25, P<0.05) when rechallenged (33). At the same time, there was no significant difference in the ORR and disease control rate (DCR) of such patients versus initial treatment which indicates that there is a promising potential of efficacy (33). This opinion was supported by the study of Gobbini et al. (n=144) that observed that patients who withdrew because of toxicity received a substantial improvement in mPFS and OS on rechallenge as compared to patients who withdrew because of PD (32). Also, LIST study where Godbert et al. analyzed the data of 522 patients with advanced NSCLC indicated that 46 patients who stopped receiving treatment due to irAEs and, in their turn, were reintroduced to receive nivolumab reported a 6-month PFS rate of 40.9% (95% CI 26.5-54.8) and a DCR of 43.2% (95% CI 28.3-59.0) (34). These were much higher than those of 197 patients who dropped out as a result of PD or other non-toxic causes (6-month PFS: 24.1; DCR: 40.2) (34). This suggests that patients with a history of irAEs may have developed effective anti-tumor immune responses and can achieve durable disease control without efficacy attenuation when rechallenged with similar agents, making them a favorable population for rechallenge.

#### 3.1.3 Clinical decision-making

For some patients, the discontinuation of initial ICI treatment is not due to explicit PD or severe irAEs but rather based on comprehensive clinical judgment and decision-making by the physician. This may include pausing treatment after completing a predetermined course, discontinuing therapy after achieving long-term remission, or other non-disease progression/non-toxicity reasons (e.g., patient preference). Such discontinuations often reflect a relatively indolent tumor biology and a more sustained immune response potential. A meta-analysis by Feng et al. of 17 studies involving 2100 NSCLC patients found that patients who discontinued due to clinical decision-making had significantly better mPFS after rechallenge compared to those discontinuing due to toxicity or PD (not reached vs. 5.2 months vs. 2.1 months, p<0.0001) (35). Similarly, Ramadoss et al.’s analysis of 45 patients with advanced Merkel cell carcinoma also indicated that ICI rechallenge is a feasible treatment strategy regardless of whether initial discontinuation was due to patient preference or toxicity (36). Other studies have reached similar conclusions (32, 37). This suggests that patients who discontinue due to clinical decision-making may retain sensitivity to ICI therapy. Therefore, maintaining the original ICI mechanism during rechallenge may be considered to re-stimulate a durable anti-tumor immune response.

### 3.2 Depth of initial treatment response

The depth of response to initial ICI therapy is a well-established critical predictor of efficacy upon rechallenge. Various researchers show that there is a strong positive relationship between the longer time of progression-free survival (PFS_1_) during the initial ICI treatment and the better prognosis following rechallenge. This correlation is related to the intrinsic immunogenicity of the tumor and the development of the anti-tumor immune memory. As an example, Yan et al. divided 165 patients, who received ICI rechallenge because of locally advanced or metastatic NSCLC, into a resistant group (PFS_1_ < 6 months, n=51) and a responder group (PFS_1_≥6 months, n=114) (40). They established that the ORR of the responder group was 17.6 compared to 7.0 of the resistant group (P = 0.038) (40). The Kaplan-Meier analysis of survival showed further that the median of PFS after second ICI treatment (PFS_2_) among the responders was 5.91 months (95%CI: 5.29-6.54), which was much higher than 3.68 months (95%CI: 2.46-4.90) in the resistant group (P = 0.014) (40). The multivariate Cox regression analysis also confirmed that initial ICI response is an independent predictor of PFS_2_ (P = 0.015) (40). Specifically, the risk of disease progression in the resistant group was approximately 56% higher than in the responder group (HR = 1.56, 95%CI: 1.09-2.24). These results clearly indicate that a longer PFS_1_ during initial immunotherapy often predicts better rechallenge outcomes (40). Importantly, this efficacy advantage does not come at the cost of safety, as both groups of patients showed no significant difference in the incidence of any-grade or Grade 3–4 adverse events (any-grade TRAEs: resistant group 68.6% vs. responder group 60.5%, P = 0.319) (40). Therefore, PFS_1_’s predictive value for rechallenge efficacy appears to be independent of safety considerations.

Similarly, Jia et al.’s retrospective study of 217 patients with advanced gastric cancer observed a consistent trend. Patients with a first-line PFS_1_ ≥ 6 months experienced significantly improved mOS (15.1 vs. 7.4 months) and mPFS (8.0 vs. 3.2 months) during second-line treatment (41). Furthermore, Vauchier et al.’s study of 45 patients with metastatic renal cell carcinoma (mRCC) supported this view, finding that patients with PFS_1_ > 6 months exhibited better rechallenge PFS, and multivariate analysis indicated that PFS_1_ > 12 months was significantly associated with a lower risk of disease progression (HR = 0.25, 95% CI 0.08-0.84; p = 0.07) (42). These findings suggest that a sustained effective response may provide the immune system with sufficient time to recognize and memorize tumor antigens, and that patients achieving long-term disease control may have a tumor microenvironment inherently more favorable to immunotherapy or possess stronger immunogenicity. Based on this, the NIVO RETURNS prospective trial for gastric cancer was designed with PFS_1_ ≥ 6 months as a key selection criterion (61). Moreover, multiple studies in solid tumors have observed similar patterns between PFS_1_ and rechallenge efficacy (46, 62–64). Crucially, as PFS_1_ further lengthens, patient benefits show an increasing trend. Feng et al.’s systematic review and meta-analysis, pooling 17 clinical datasets, clearly demonstrated that patients with PFS_1_ exceeding 2 years had significantly better ORR, DCR, and mPFS after rechallenge compared to those with PFS_1_ less than 1 year (ORR: 35.0% vs. 9.8%, p = 0.03; DCR: 85.0% vs. 49.0%, p = 0.007; mPFS: 12.4 vs. 3.0 months, p<0.001) (35). A retrospective analysis from Zhejiang Provincial Cancer Hospital (n=104) further confirmed that NSCLC patients with PFS_1_ ≥ 12 months had significantly prolonged mPFS (9.2 vs. 3.4 months, P<0.001) and mOS (25.5 vs. 10.7 months, P = 0.006) after rechallenge (43). This implies that an extended PFS_1_ might also imply that there is increased tumor sensitivity to immunotherapy or there is a deeper immune memory produced, which increases survival benefits upon rechallenge.

According to the growing number of facts, the predictive value of PFS_1_ is generally accepted. The systematization of available evidence provided in the Italian Delphi expert consensus study, which was conducted by Colonese et al., was very clear that the minimum threshold at which ICI rechallenge needs to be considered was at least 6 months of disease control (i.e., PFS_1_≥ 6 months) (44). This opinion was further supported by the analysis of Levra et al., which used large-scale rechallenge data (n=1517) based on real-world French data (PFS_1_≥ 6 months) and concluded that this single predictor of overall survival after rechallenge (OS_2_) (45). The OS_2_ hazard ratio of patients with PFS_1–_6 months compared to patients with PFS_1_ less than 3 months was significantly lower (responder group: 0.14-0.25, rechallenge group: 0.10-0.33) (45). This also justifies the application of this threshold as one of the major criteria in choosing beneficial populations to be rechallenged (45). To sum up, firstly, the initial research has made ICI treatment PFS_1_ a significant part of the clinical decision-making process in different types of cancer and expert consensus in assessing the probability of rechallenge benefit that provides strong evidence of accurately identifying potential beneficiaries.

### 3.3 Treatment-free interval

In addition to the depth of initial response (PFS_1_), length of treatment-free interval (TFI) has been found to be another crucial predictive variable on whether ICI rechallenge is effective. TFI is described as the period of time between the disappearance of the former ICI therapy and the beginning of the new ICI therapy. Thorough evaluation of the available clinical evidence will help to understand that a long-enough TFI can grant the immune system with the required period of recovery and remodeling, thus defining the final effectiveness of rechallenge. Several reports have also supported a high correlation between an increased TFI and excellent clinical outcomes following rechallenge. A study of the Japan Lung Cancer Group (NJLCG) on rechallenge of 38 patients with advanced NSCLC revealed that the ORR and DCR of patients with a TFI over 11.9 months rechallenge was 21.1 and 63.2, respectively. By contrast, none of the patients had a longer TFI (>11.9 months) responded (ORR 0%) and only had a DCR of 31.6% (47). The multivariate analysis also confirmed that the TFI 11.9 months and above is an influential independent prognostic factor (HR = 0.33, 95% CI = 0.15-0.76, p=0.009) (47). More to the point, the rewards gained by a longer TFI were embodied not in the initial response rate but also in sustained control over the disease. It was found that patients with TFI > 11.9 months had significantly long PFS (mPFS: 4.3 months vs. 1.8 months) (47). Even though the mPFS of the overall cohort was as low as 2.5 months, the 1-year and 2-year PFS rates were 13.8% (95% CI: 5.1-26.8%), and the PFS curve showed some sort of long-tail plateau effect (47). This indicates that patients with a higher TFI are able to attain long-term disease control, this may be because of reversal of T-cell exhaustion and re-establishing of anti-tumor immune responses (47, 65).

This observed trend has been continually confirmed in different solid tumors. The study conducted by Owen et al. (n=147) in melanoma showed significantly varied rechallenge (48). In those patients who had progressed under adjuvant therapy with PD-1 (n=104), the ORR of a second round of PD-1 monotherapy was 0% (0/6) (48). Conversely, in 32 patients suffering a relapse following adjuvant therapy and having a longer TFI (median time to relapse post-discontinuation: 5.5 months), 2 of 5 patients (40%) residing at PD-1 rechallenge responders (48). This points to the possible usefulness of TFI in PD-1 rechallenge, although the pretreatment modality and tumor biology could potentially have an impact. The prognostic value of TFI was further explained by a US real-life study that dealt with 334 patients with head and neck squamous cell carcinoma (49). It was found that patients who had a long TFI (median 6.4 months) had an improved mOS following rechallenge (15.7 months vs. 9.9 months), and an increase in the survival benefit was approximately 1.6-fold, compared to the patients who had a short TFI (median 5.8 months) (49). This implies that a higher TFI can be used to predict improved response to rechallenge (49). The predictive value of TFI was systematically tested by Zhao et al. in a large-sample retrospective study on 352 patients with NSCLC by using a multivariate model (46). The researchers established that TFI ≤ 6 months is an independent risk factor of shortened PFS and OS after rechallenge, which significantly risked the progression and mortality of the disease by 83% (HR = 1.83, 95%CI: 1.39-2.40, P<0.001) and 80% (HR = 1.80, 95%CI: 1.33-2.44, P<0.001), respectively (46). Summarily, a long enough TFI is an essential requirement to successful rechallenge of ICI. It has the potential to help the body leave the immunosuppressive condition caused by previous treatment and establish the conditions under which the good anti-tumor immune responses would be restored. Thus, the combination of TFI and PFS_1_ into clinical decision-making will assist in being more precise about the populations that are likely to benefit more with rechallenge strategies.

### 3.4 Resolution criteria and timing for irAEs rechallenge

The incidence and intensity of irAEs have a significant impact on the decision on rechallenge of ICI. Although previous irAEs may signify the sensitivity of a patient to immunotherapy, it simultaneously predicts the risks that may occur in case of further treatment. Hence, thorough evaluation of irAEs is an essential requirement towards the balancing of risks and benefits of rechallenge. The criteria according to which irAEs should be resolved are not universal, rather, they require a subtle evaluation depending on the type and the severity of the irAE. In current consensus guidelines, to be considered, irAE symptoms and lab parameters must have returned to CTCAE Grade ≤ 1 to be considered (66–70). As well, corticosteroid dose must be reduced to 10 mg/day prednisone equivalent and below. This is a high standard, which is achieved to guarantee that any underlying immune inflammation is sufficient covered in order to stop re-activation of irAEs during rechallenge.

In the majority of low-grade irAEs (Grade 1-2), e.g., mild rash or endocrine dysfunction, rechallenge can be considered to be possible in case of the symptoms having improved to CTCAE Grade ≤ 1 and the general condition of the patient being stable. Several studies support this approach. Wang et al. retrospectively studied 595 lung cancer patients, among whom 35 developed ICI-related colitis (66). For 19 patients who underwent rechallenge after their colitis resolved to ≤ Grade 1 and corticosteroids were tapered, the mOS was 36 months, which was significantly superior to the 32 months observed in the non-rechallenge group (P = 0.026) (66). This finding suggests that for well-controlled mild-to-moderate irAEs, cautious rechallenge strategies can not only re-initiate anti-tumor immunity but also lead to significant survival benefits. Furthermore, Yang et al. retrospectively analyzed 81 advanced lung cancer patients and found that among 40 who underwent ICI rechallenge after irAE-related discontinuation, the DCR reached 75% (50). While 9 patients (22.5%) experienced irAEs recurrence, most recurrent irAEs were Grade 1-2 (50). This study also notably found that the median time to irAEs onset after ICI rechallenge was 21 days, which was earlier than during initial ICI treatment (50). Consequently, the authors emphasized the critical need for earlier and closer monitoring of irAEs during ICI rechallenge (50). Similarly, Hwang et al. conducted a systematic review and meta-analysis of 1856 ICI hepatitis patients, showing that approximately 40% (95% CI: 30%-51%) of patients underwent ICI rechallenge after hepatitis resolution or improvement to Grade 1 (51). The hepatitis recurrence rate was approximately 22% (95% CI: 15%-30%), and crucially, the severity of recurrent hepatitis was generally lower than the initial event (51). These studies, along with others (14, 50, 52, 71–74), collectively demonstrate the good tolerability of ICI rechallenge after complete resolution of irAEs for patients whose initial treatment was discontinued due to irAEs.

However, high-grade irAEs (≥ Grade 3) pose a more formidable challenge for rechallenge. Expert guidelines from SITC, ESMO, and ASCO unanimously advise extreme caution when reintroducing ICI treatment in patients with a history of Grade 3–4 irAEs, due to the potential risks of severe toxicity (75–77). Multiple retrospective studies indicate that for specific organ-specific Grade 3–4 irAEs, if toxicity has fully resolved to its best state and treatment strategies are adjusted (e.g., de-escalating from combination therapy to anti-PD-1 monotherapy), patients may still benefit from cautious rechallenge and experience significant tumor response (53, 55, 78, 79). Nevertheless, the feasibility varies significantly by organ. For renal toxicity (ICPi-AKI), Cortazar et al.’s study of 138 patients who experienced ICPi-AKI found that about 23% had recurrent AKI after rechallenge, and the median rechallenge interval for recurrent AKI patients was significantly shorter than for non-recurrent patients (56). This is an indication that premature rechallenge can augment the chance of reoccurrence, consequently necessitating a higher degree of prolonged periods of observation and closer observance of the renal functioning. In the case of pneumonitis, Koyauchi et al. and Li et al. found that despite the complete resolution of symptoms, as well as the use of corticosteroids, rechallenge is a severe risk of observing an event again, and in many cases, a recurrent event is accompanied by the implementation of lower grades of adverse events or even death, so extreme caution is needed (8, 71). In the case of ICI-related cardiotoxicity, because of the high percentage of fatality (up to 38% to 46) and high risk of cardiovascular events, the present clinical guidelines tend to advise irreversible withdrawal of associated ICI therapy, as it is an absolute contraindication to rechallenge (54). Thus, high-grade irAEs rechallenge decisions need to be made with the utmost caution with a multi-dimensional, individualized approach that transcends on grading with an organ specific risk, depth of resolution, and mitigation strategy.

### 3.5 Patient baseline characteristics and disease progression patterns

Patient baseline characteristics and patterns of disease progression are central to consider, in terms of ICI rechallenge, to determine the feasibility of treatment and prognosis. Not only do these factors provide a reflection of the general health and tolerance of the patient to therapy, they provide a hint on whether the tumor is sensitive to immunotherapy or not.

#### 3.5.1 Performance status and prognosis

Eastern Cooperative Oncology Group (ECOG) Performance Status (PS) score is the typical way of assessing patient performance status. Multiple studies have confirmed its close relationship with the efficacy and safety of ICI rechallenge. Numerous retrospective studies consistently indicate that an ECOG PS score ≥ 2 (i.e., poor performance status) is a strong independent predictor of adverse prognosis after rechallenge (32, 40, 41, 49, 57–60, 80). These patients experience significantly increased risks of disease progression and death. Musaelyan et al.’s analysis of ICI rechallenge in 52 patients with advanced NSCLC showed that an ECOG PS score ≥ 2 was negatively correlated with shorter PFS (HR = 3.41, 95% CI: 1.28-9.05; P = 0.013) (40). This indicates that patients with poor performance status experience significantly reduced disease control duration after rechallenge. Yan et al.’s study of 224 patients with locally advanced or advanced NSCLC also found that compared to patients with good performance status (ECOG PS 0), patients with ECOG PS score ≥ 2 after rechallenge had significantly lower DCR for second-line therapy (71.7% vs 45.5%, P = 0.032) and significantly shorter PFS (7.98 months vs 3.68 months, P = 0.015) (40). Zhang et al.’s study of 329 patients with esophageal squamous cell carcinoma further confirmed this trend, showing that an ECOG PS score ≥ 2 was independently associated with worse PFS (HR = 2.56, p=0.003) and OS (HR = 1.95, p=0.032) (58). These findings taken together prove that the better the performance status (lower ECOG/PS score, usually ≤ 1), the higher the probability that the patient will benefit from ICI rechallenge.

The multifaceted correlation between performance status and ICI rechallenge efficacy is due to several factors. Patients with good performance status in general are those with better organ function reserves and can tolerate the potential toxicities associated with ICI rechallenge. Their immune systems are often more intact and thus may have a better response to immune checkpoint inhibitors, which may promote a more effective anti-tumor immune response (32, 57, 58). On the opposite side, patients with poor performance status may have more severe immune dysregulation (e.g., impaired effector T-cell function), which can affect the effectiveness of immunotherapy (32, 57, 58). Therefore, the ECOG PS score should be an important criterion for screening patients for ICI rechallenge and patients with an ECOG score of 2 or better should be cautiously evaluated to avoid unnecessary toxicity and ineffective treatment.

#### 3.5.2 Patterns of disease progression and tumor burden

In addition to general patient performance status, the specific pattern of disease progression and tumor burden also are critical factors in determining the efficacy of ICI rechallenge. Patients who get oligoprogression after initial ICI treatment, i.e. few lesions progress or new lesions are seen while most are controlled, may do better with rechallenge. Liu et al.’s study of 86 patients with Extensive-Stage Small Cell Lung Cancer (ES-SCLC) undergoing cross-line immune rechallenge found that patients with progression manifests as only new lesions had significantly better mOS than those with progression limited to primary lesions (17.73 months vs. 9.17 months) (81). Furthermore, the absence of liver metastases also was found to be a good prognostic indicator (mOS: 14.23 months vs. 11.67 months) (81). This progression pattern may suggest that the tumor retains some immune sensitivity, allowing local therapies (such as radiotherapy) to clear progressing lesions, thereby restoring systemic response to ICI and even inducing an “abscopal effect” that remodels the tumor immune microenvironment (82).

Tumor burden, particularly the number and location of metastatic sites, also significantly impacts rechallenge efficacy (42, 83–85). Shang et al. included 125 ES-SCLC patients from the IMpower133 cohort and 161 from the Shanzhong cohort, all of whom experienced disease progression after first-line ICI treatment (84). They found that patients with fewer than 4 metastatic sites had significantly prolonged OS after rechallenge (HR: 0.457; 95% CI: 0.256-0.817; P = 0.008) (84). The authors suggested that the mechanism might be that lower tumor burden is generally associated with a more favorable tumor microenvironment, such as reduced M2 macrophage infiltration and increased T-cell infiltration, thereby enhancing anti-tumor immune responses (84). Additionally, the presence or absence of liver metastases is an important prognostic factor. Shi et al.’s study of 28 ES-SCLC patients receiving ICI rechallenge indicated that baseline liver metastases were an independent factor affecting OS (HR = 0.13, 95%CI: 0.03-0.72, p=0.02), with a lower HR value indicating a poorer prognosis associated with the presence of liver metastases (85). The authors believe that the liver, as a complex organ, may contribute to stronger systemic immunosuppression, or its metabolic function may affect the distribution and action of immune drugs, thereby weakening the anti-tumor effect (85). In Vauchier et al.’s study of 45 patients with metastatic renal cell carcinoma undergoing ICI rechallenge, univariate analysis clearly showed that high tumor burden (specifically, an increased number of metastatic sites and the presence of liver metastases) was associated with significantly shorter PFS after rechallenge (42). These factors together prove the patient with advanced disease, high tumor burden and poor overall health status have worse prognosis with ICI rechallenge that can be due to stronger tumor immune evasion mechanisms or a complex microenvironment. Therefore in the decision of continued ICI treatment, the location of metastatic lesions, in particular the presence of liver metastases, should be important prognostic indicators.

In summary, a complete assessment of patient attributes and previous response to therapy is essential in ICI rechallenge. This is a comprehensive framework that combines factors such as PFS_1_ at baseline, TFI, reasons for initial discontinuation (irAEs, PD or clinical decision-making) of ICI, performance status of the patient (ECOG PS score) and specific patterns of disease progression (tumor burden and metastatic sites). Clinicians are encouraged to synthesize these multi-dimensional clinical features in order to make individualized decisions in order to maximize benefits to patients while proactively avoiding risks. Future research efforts should be aimed towards the development of more sophisticated dynamic biomarkers and predictive models in order to provide information for better patient selection and more precise guidance in complex clinical settings.

## 4 ICI rechallenge strategies

**Figure 2:**
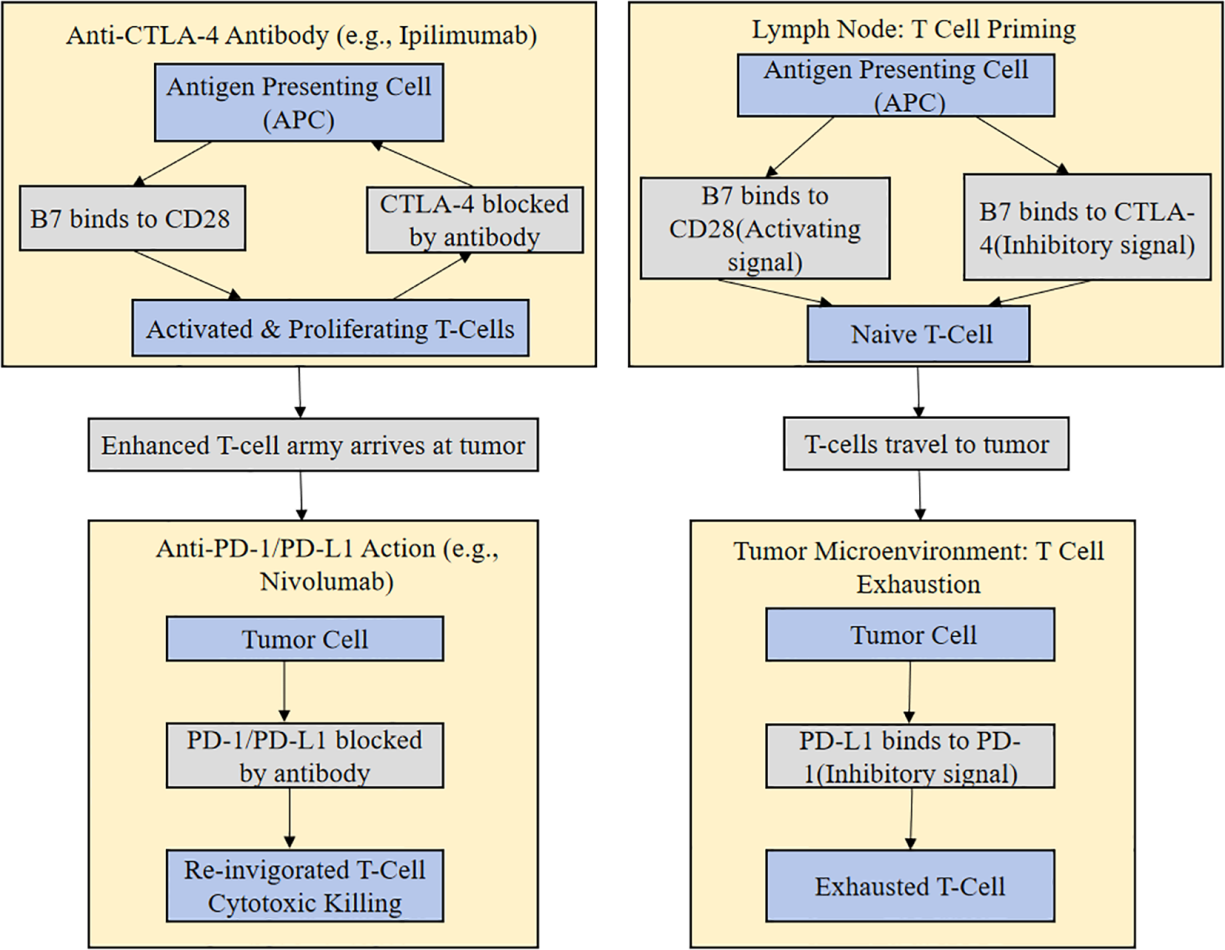
Mechanisms of action of different immune checkpoint inhibitors and rationale for combination therapy. This schematic summarizes the mechanisms of action of immune checkpoint inhibitors and the rationale for combination therapy in the rechallenge setting. The figure compares the sites of action for anti-CTLA-4 versus anti-PD-1/PD-L1 inhibitors at different stages of T-cell activation (priming vs. effector function). It further demonstrates the use of ICI with chemotherapy, anti-angiogenic agents, or local radiotherapy to synergize in order to increase the efficacy of rechallenge due to the release of tumor antigens, tumor microenvironment modification, or to overcome specific resistance mechanisms.

Following initial failure of ICI treatment, often monotherapy rechallenge is limited by complex mechanisms of resistance. Therefore, combinations strategies that have synergistic effects of remodeling the tumor microenvironment are pivotal to overcome the resistance. The synergistic mechanisms underlying these various combination strategies aiming at targeting the effects of ICI at different stages of the immune response schematically represented at **Figure 2** are considered pivotal. By means of chemotherapy, anti-angiogenic drugs, or local radiotherapy, these approaches can release tumor antigens, reverse immunosuppression, and eliminate resistant clones, thereby synergizing with ICI to create favorable conditions for successful rechallenge (86–92). A comprehensive overview of the objective response rates, median progression-free survival, and safety profiles for these major combination strategies, as well as ICI monotherapy, across various representative cancer types, is presented in **Table 2**.

**Table 2:** Efficacy and safety of major combination strategies for ICI rechallenge.

Strategy	Representative Cancer Type(s)	Objective Response Rate (ORR, %)	Median PFS (mPFS, months)	Safety Profile	Representative Study
ICI Monotherapy Rechallenge	Gastric Cancer, Melanoma, NSCLC	15.0 – 30.0	5.4 – 8.0	Manageable; irAEs recurrence risk depends on history.	(62, 93–98)
ICI + Chemotherapy	NSCLC, Gastric Cancer	20.0 - 35.0	6.7 - 12.0	Manageable, consistent with chemotherapy toxicity.	(88, 99–101)
ICI + Anti-angiogenesis	ESCC, NSCLC, Nasopharyngeal Carcinoma	~20 – 25	5.6 – 7.9	Generally manageable; monitor class-specific events (e.g., hypertension).	(88, 102–104)
ICI + Local Radiotherapy	NSCLC (Oligoprogressive)	~20	6.6 - 9.0	Risk of radiation pneumonitis (~15%).	(40, 82, 91)
Dual ICI Combination	RCC, HCC, Melanoma	10.3 – 29.0	3.1 - 4.0	Substantially increased risk of irAEs (≥Grade 3 irAEs ~25%).	(105–109)
Triple-Drug Combination	NSCLC, Gastric Cancer	Variable (Reported range: ~20 – 30)range: ~20 – 30)	Variable (Reported range: 4.7 – 9.8)	Variable, potential for increased toxicity.	(41, 100, 110, 111)

### 4.1 Chemo-ICI combination

Chemotherapy combined with ICI (Chemo-ICI) is one of the most commonly used strategies for ICI rechallenge. Chemotherapeutic agents directly kill tumor cells and induce immunogenic cell death, exposing tumor antigens and eliminating immunosuppressive cells, thereby improving the tumor microenvironment and enhancing immunogenicity (86). Crespi et al. described 20 patients with unresectable locally advanced NSCLC (Stage III) who failed durvalumab consolidation and benefited from immune-chemotherapy rechallenge after PD (mPFS: 12.0 months, 95% CI: 1.6-22.4), which was superior to chemotherapy alone (4.1 months, P = 0.009) and ICI alone (2.1 months, P = 0.009) (99). Additionally, Guo et al. reported that among 85 patients with advanced gastric cancer, 50 patients who progressed after first-line immunotherapy underwent ICI-chemotherapy rechallenge, with an ORR and DCR of 20.0% and 78.0%, respectively, and an mPFS of 6.7 months, which was superior to conventional second-line chemotherapy (100). Similar results have been observed in other cancer studies (38, 88, 101, 112). This further indicates that chemo-ICI combination has good synergistic efficacy across various cancer types in rechallenge treatment. However, the benefits of chemo-ICI rechallenge are not uniform across studies. Tompkins et al. studied 532 NSCLC patients and found that the mOS (7.6 months) and mPFS (4.6 months) in the immune-chemotherapy combination group (n=49) did not show statistically significant superiority over chemotherapy alone (n=436) or immune monotherapy (n=47) (113). The researchers noted that this might reflect the more advanced disease characteristics of patients selected for chemo-ICI rechallenge, who had a higher proportion of Stage IV disease at baseline and shorter first-line PFS. This suggests that clinicians might tend to select combination strategies for patients with more aggressive tumors. However, this strategy did not result in statistically significant survival improvement in the study by Tompkins et al. (113). Therefore, the benefits of combination chemotherapy may be limited to specific patient populations, especially those with high tumor burden and aggressive tumors, and may require more stringent patient selection criteria for efficacy.

### 4.2 Anti-angiogenesis-ICI combination

The combination of anti-angiogenic drugs with ICI is another highly anticipated and promising direction in ICI rechallenge strategies. Anti-angiogenic drugs normalize tumor vasculature and improve T-cell infiltration, thereby synergizing with ICI to significantly enhance anti-tumor immune efficacy (86). Multiple studies have confirmed the potential of this strategy (43, 102, 103, 114–118). In advanced esophageal squamous cell carcinoma, Hong et al. included 110 patients for analysis (102). The results showed that anlotinib combined with PD-1 inhibitors for rechallenge achieved an mOS of 11.1 months, an mPFS of 5.6 months, an ORR of 19.1%, and a DCR of 69.1%. Meanwhile, the safety profile of this combination regimen was manageable, with a Grade ≥ 3 treatment-related adverse event (TRAE) incidence of 10.0% (102). A retrospective study analyzed the efficacy of ICI monotherapy or combination rechallenge after first-line ICI plus chemotherapy in 154 patients with advanced NSCLC (88). The results indicated that the mPFS of the ICI-anti-angiogenic combination therapy group was the longest, reaching 5.7 months (compared to 3.6 months for ICI monotherapy, 3.2 months for chemotherapy-ICI, and 2.9 months for chemotherapy-ICI-anti-angiogenesis combination), with a statistically significant difference (p=0.0086) (88). Similarly, in nasopharyngeal carcinoma, Jiang et al.’s analysis of 145 patients further confirmed that compared to chemotherapy alone (n=97), the mPFS of the immune-anti-angiogenic combination therapy group (n=48) was significantly prolonged (7.9 months vs 4.4 months), and the DCR was significantly improved (83.33% vs 64.95%), with comparable safety (104). Furthermore, multivariate Cox regression analysis showed that combination therapy was significantly associated with improved PFS (HR = 0.363, 95%CI 0.228-0.578, p < 0.001) (104). However, the success of combination strategies is not universally applicable, and their efficacy may be highly dependent on specific treatment lines, drug sequences, and tumor types. For example, in metastatic clear cell renal cell carcinoma, Georges Gebrael et al.’s real-world study (n=348) showed that after first-line PD-1/L1 inhibitor failure, second-line cabozantinib combined with PD-1/L1 inhibitor (n=68) did not show superior efficacy compared to cabozantinib monotherapy (n=280) and did not bring additional survival benefits (119). This finding is consistent with the results of the Phase III CONTACT-03 clinical trial (119). This suggests that not all combinations of anti-angiogenic drugs and ICI are successful in the context of immune resistance, and their efficacy may be influenced by tumor type, prior treatment exposure, and specific drug combinations, thus requiring more cautious and individualized strategy selection.

### 4.3 Combination local therapy

ICI combined with local therapy is an ideal option for oligoprogression. Local therapies (such as radiotherapy and ablation) induce immunogenic cell death, releasing tumor antigens, thereby initiating anti-tumor T-cell responses. This process can enhance the immunogenicity of the tumor microenvironment, synergize with immune checkpoint inhibitors, overcome local resistance, and induce systemic “abscopal effects” (40). Hirano et al.’s study showed that in 20 NSCLC patients with ICI-resistant oligoprogression, adding local radiotherapy to the original ICI regimen significantly prolonged mPFS to 9.0 months, which was significantly better than 1.6 months in the non-local therapy combination group (P = 0.02) (82). Yan et al. analyzed 224 NSCLC patients receiving second-line treatment, among whom 56 patients used radiotherapy combined with ICI for rechallenge (40). Their ORR (21.4% vs 8.3%, P = 0.008) and DCR (78.6% vs 60.7%, P = 0.015) were significantly superior to the non-radiotherapy combination group, and mPFS was also significantly prolonged (6.64 months vs 4.04 months, P = 0.036) (40). Notably, there was no significant difference in the incidence of any-grade TRAEs between the two groups, indicating that combined local radiotherapy did not significantly increase toxicity under specific conditions (40). In addition, Yamamoto et al. also observed in a study of 36 SCLC patients that 4 patients who received local ablative radiotherapy combined with ICI rechallenge achieved an mOS extension to 39.3 months, with manageable safety (120). Similarly, Zhao et al.’s study of 109 patients with esophageal squamous cell carcinoma also showed that the median second-line OS for ICI combined with chemoradiotherapy was 11.2 months (95% CI: 9.4-19.0), which was superior to historical controls of 6.2-8.4 months (121). Other studies have also reported similar results (122). Although combined local therapy shows good efficacy, its potential risks should not be overlooked. Hirano et al. found that the incidence of Grade 1–2 radiation pneumonitis was about 15% (82). Therefore, combined local immunotherapy is best suited for patients with ECOG 0–1 performance status and without extensive pulmonary fibrosis. This strict patient selection criterion aims to minimize potential toxicity and ensure treatment safety and efficacy.

### 4.4 Dual ICI inhibitor combination

In ICI rechallenge strategies, beyond single-agent ICI therapy, combination therapies, especially the simultaneous use of two ICI inhibitors with different mechanisms, has become a promising direction. Given that different ICI targets (such as PD-1/PD-L1 and CTLA-4) exhibit significant complementarity in activating T-cell immune responses at different stages and mechanisms (e.g., CTLA-4 primarily regulates T-cell initiation and proliferation, while PD-1/PD-L1 primarily affects T-cell effector function in the tumor microenvironment), this combination strategy aims to synergistically overcome immune suppression in the tumor microenvironment through multiple pathways, thereby significantly enhancing anti-tumor effects and prolonging patient survival (105).In mRCC, Gul et al.’s study showed that after prior PD-1 pathway inhibitor treatment failure, salvage treatment with ipilimumab (CTLA-4 inhibitor) combined with nivolumab (PD-1 inhibitor) achieved an ORR of 20%, an mPFS of 4 months, and some patients who initially failed treatment still benefited (105). Additionally, Ravi et al.’s study also demonstrated that 22 mRCC patients who received dual ICI inhibitor combination therapy achieved an ORR of 25% (106). Yonemoto et al.’s multicenter retrospective analysis of 68 patients with advanced hepatocellular carcinoma found that patients who underwent rechallenge with durvalumab combined with tremelimumab achieved an ORR of 10.3%, a DCR of 58.8%, and an mPFS of 3.1 months (107). Other studies have also observed similar results (108, 123, 124). Papathanassiou et al.’s (including 10 studies, 500 mRCC patients) and Cao et al.’s (including 60 studies on advanced solid tumors) meta-analyses also showed that patients receiving dual ICI rechallenge can achieve good efficacy (101, 125). However, while this strategy brings stronger immune activation, it may also increase the risk of irAEs. Cao et al.’s meta-analysis pointed out that although combining different ICI mechanisms (such as anti-CTLA-4 with anti-PD-1) can improve ORR, the incidence of Grade ≥ 3 irAEs can be as high as 25.4% (95% CI: 17.8%, 33.7%), significantly higher than single-agent anti-PD-(L)1 therapy (8.1%) (101). It is particularly noteworthy that this risk is more pronounced when ipilimumab is used at higher doses, with Grade 3–4 irAEs incidence increasing from 22% to 36% (123). Therefore, although combination strategies can overcome resistance through potent immune activation, clinical decisions must carefully weigh the survival benefits against the associated increased toxicity risks.

### 4.5 Triple-drug combination strategies

Within the landscape of ICI rechallenge, triple-drug combination regimens represent a cutting-edge frontier in overcoming treatment resistance. This strategy is based on the combination of an ICI and two distinct standard anti-tumor interventions, such as chemotherapy, radiotherapy or targeted therapy. The big picture is to use the multiple pathway synergy for a more powerful anti-tumor effect (110, 126). However, efficacy and safety profiles of triple-drug combinations show a high degree of variability for different tumor types and treatment situations. For example, Feng et al. found high DCR, 86.5%, of 111 patients with NSCLC were treated with ICI plus chemotherapy and anti-angiogenic treatment with no significant risk of immunotoxicity (110). Similar results were confirmed by the study of Musaelyan et al. (n=43) (126). In addition, the study conducted by Geng et al. showed that 23 patients, who received a combination of immune checkpoint inhibitors, anti-angiogenic agents, and immunomodulators, had an ORR of 21.7%, a DCR of 73.9%, and a mOS of 9.8 months, with a manageable safety profile (111). These data are cumulatively evidence for the potential usefulness of triple drug regimens in certain cohorts of patients. On the other hand, there are studies which have shown conflicting or small benefits. In the context of gastric cancer, for example, a retrospective study of 217 patients with advanced disease by Jia et al. found that the combination of ICI, chemotherapy and anti-angiogenic drugs increased mOS to 11.5 months compared with dual drug regimens (95% CI 9.3-13.7, p=0.032) (41). However, on the contrary, the retrospective analysis by Guo et al. (n=85) suggested that the use of a third drug led to a decrease in mPFS from 6.7 months to 4.7 months with a significant increase in the risk of TRAEs (100). This great inconsistency strongly argues that the efficacy of triple-drug regimens is highly dependent on the particular type of the tumor, the exact combination of agents and strict selection of patients. The theoretical advantages in overcoming resist may be offset by cumulative toxicities and cause complex clinical difficulties in their implementation. Consequently, the role of triple drug combination strategies in ICI rechallenges remain largely investigational and settings need much more high-quality prospective studies that will ensure the optimal tumor types, drug combinations, patient selection criteria, and dose optimization strategies for maximal efficacy with the ability to effectively manage potential risks.

### 4.6 Strategies for immune checkpoint inhibitor selection

In the context of ICI rechallenge, the choice of immune agents mainly focuses on two different approaches: rechallenge with the same mechanism of action (MOA), or the switch to a different MOA. Each approach has its own background in terms of theoretical rationales, clinical evidence, and benefits and risks.

#### 4.6.1 Rechallenge of same mechanism of action

Rechallenge with an ICI based on the same MOA has been given secondarily on the premise that the immune system has a memory response to the previously administered drug. Even after disease progression, reintroducing an ICI with the identical mechanism is hypothesized to reactivate this immune memory, thereby sustaining tumor control. Clinical data support this concept in certain scenarios. Zhang et al. demonstrated in a study of 60 patients with advanced gastric cancer that those rechallenged with the same PD-1 monoclonal antibody showed significantly longer mPFS compared to patients receiving a different PD-1 antibody (3.5 months vs. 1.9 months, P = 0.006) (62). Furthermore, the safety profile in the same-drug group was manageable, with grade 1–2 and grade 3–4 adverse event rates of 83.3% and 35.0%, respectively (62). Li et al.’s retrospective analysis of 80 advanced gastric cancer patients further revealed that the same PD-1 inhibitor regimen (n=56) led to a significantly higher DCR (51.8% vs. 29.2%, P = 0.062) and extended mPFS (162 days vs. 75 days, P = 0.001) and OS (312 days vs. 166 days, P = 0.027) compared to different regimens (93). Multivariate analysis further confirmed the association between the same regimen and longer PFS (HR = 0.467, P = 0.008) and OS (HR = 0.508, P = 0.027) (93). Similar findings were reported by Whitman et al. in 19 melanoma patients treated with the same anti-PD-1 monotherapy, achieving 1-year and 2-year OS rates of 100% and 83%, respectively (94). Other studies have also corroborated these observations (35, 95–97, 127–130). Collectively, this evidence suggests that in specific tumor types, maintaining the initial ICI mechanism for rechallenge is a viable and promising strategy.

However, the efficacy of same-MOA rechallenge is not universally applicable and can be influenced by various factors. Some research indicates that tumors may develop resistance by upregulating regulatory immune factors, rendering subsequent rechallenge with the same drug ineffective (65). For instance, Horisaki et al.’s study in stage IV malignant melanoma patients revealed a 0% ORR and a mere 8.8% DCR in 34 patients rechallenged with anti-PD-1 antibodies, with a median PFS of only 2.1 months (131). This was significantly shorter than in the BRAF/MEK inhibitor rechallenge group (ORR: 14.3%, DCR: 50.0%, mPFS: 3.0 months) (P = 0.025), suggesting the development of resistance to the PD-1 pathway (131). Additionally, a broad retrospective analysis of over 24,000 irAEs cases by Dolladille et al. found that the irAEs recurrence rate for anti-CTLA-4 monotherapy (47.4%) and combination therapy (43.5%) rechallenge was higher than for anti-PD-1 or anti-PD-L1 monotherapy (28.6%) (14). In summary, same-MOA ICI rechallenge can offer additional clinical benefits and favorable survival outcomes in a subset of patients, particularly for PD-1 or PD-L1 monotherapy rechallenge. Nevertheless, given the intricate nature of resistance mechanisms and the current research primarily focusing on gastric cancer, melanoma, and NSCLC, further validation in other solid tumors is warranted. Crucially, patient selection based on biomarkers remains essential to identify the specific populations most likely to benefit.

#### 4.6.2 Cross-Mechanism ICI switching

When rechallenge with the same ICI mechanism proves ineffective or is not feasible, switching to a different mechanism emerges as a pivotal strategy to overcome acquired resistance. This approach is predicated on the understanding that PD-1/PD-L1 inhibitors and CTLA-4 inhibitors exert their effects at distinct stages of the immune response (e.g., T-cell priming/activation and effector function) through non-overlapping mechanisms, holding promise for overcoming resistance that developed after single-agent PD-1 inhibition (132). Several studies have demonstrated the potential of cross-mechanism switching. Weber et al. reported that among 92 melanoma patients who progressed on ipilimumab (anti-CTLA-4) therapy, switching to nivolumab (anti-PD-1) resulted in an ORR of 29% and a mOS of 20.6 months, notably without the recurrence of severe irAEs (109). Wicky et al. further corroborated this by showing that among 35 advanced melanoma patients who progressed on first-line PD-1 therapy, 65% (20 patients) who switched to a combination of CTLA-4 and PD-1 inhibitors for rechallenge achieved significantly better OS compared to those who switched to targeted therapy (HR = 0.46, 95% CI: 0.22-0.94, p=0.03) (133). This enhanced efficacy may stem from CTLA-4 inhibition increasing the diversity of anti-tumor immune responses within lymph nodes and depleting regulatory T cells in the tumor microenvironment, while PD-1/PD-L1 blockade primarily reactivates exhausted tumor-infiltrating lymphocytes locally. The synergistic effect of these two mechanisms is believed to be crucial (57, 134, 135). Takahara et al.’s research on ICI rechallenge in NSCLC patients also identified “cross-mechanism ICI switching” as a key factor for achieving disease control, with all 11 responders in their study having adopted a different ICI mechanism than their initial treatment, a statistically significant difference compared to non-responders (p=0.006) (136). Other studies have yielded similar results (48, 63, 107, 137–140). Despite its potential, cross-mechanism switching is not without limitations. The choice of switching strategy, particularly the sequence of targets, significantly impacts treatment outcomes. A prospective study by the NJLCG (NJLCG 1901, n=38) found that sequential anti-PD-L1 to anti-PD-1 treatment might offer a clinical advantage over the reverse sequence (mPFS: 4.7 months vs. 2.2 months), possibly due to anti-PD-1 antibodies’ ability to additionally inhibit the PD-L2 signaling pathway (47). However, two retrospective studies by Fujita et al. indicated that regardless of the treatment sequence, patients receiving rechallenge did not achieve partial or complete responses, with best responses being only stable disease or progressive disease, and very short PFS (mPFS between 1.9 and 2.9 months) (135, 141). The authors attributed this to the generally poor physical condition of patients, extensive prior treatment lines, and the complex resistance mechanisms developed by their tumors (135, 141). Thus, in the context of ICI progression, a good baseline performance status is considered a crucial prerequisite for ICI rechallenge.

Indeed, both aforementioned treatment strategies possess their own contexts of applicability and inherent limitations. Research indicates that while patients undergoing ICI rechallenge, irrespective of whether the same or a different type of ICI is chosen, generally experience a reduction in overall and high-grade irAEs. However, the specific treatment regimen does not appear to significantly influence the risk of irAE recurrence (33, 142–144). According to the final analysis of CheckMate 067 patients experiencing at least 80% tumor shrinkage with either CTLA-4 inhibitors (e.g., ipilimumab) or PD-1 inhibitors (e.g., nivolumab) as monotherapy were able to survive long term (145). For patients who had an initial good response to treatment but stopped therapy for reasons other than disease progression, a continuing strategy of the same mechanism ICI rechallenge may have some advantages. However, the switch to a cross mechanism strategy, while holding promise to overcome specific resistance mechanisms, can paradoxically increase the risk of irAEs (52, 101). This phenomenon is attributed mainly to the different mechanism of action of CTLA-4 and PD-(L)1 inhibitors: the CTLA-4 inhibitors activate T cells mainly in the early stages, especially in the lymph nodes, and such a wide activation may affect centrally the immune tolerance, resulting in a higher incidence of irAEs with a more widespread action on the overall regulation of the immune system (132). In contrast, PD-(L)1 inhibitors have predominantly their effects in peripheral tissues through disinhibition of cytotoxic function of T cells, thus restoring of anti-tumor immune responses and their action is relatively more confined to the tumor microenvironment (132). The different mechanisms and resulting patterns of adverse events of these two classes of inhibitors are noteworthy. A pharmacodynamic study has shown that following treatment with an antibody against PD-1, over 70% of PD-1 receptors expressed on peripheral blood T cells are occupied for >2 months (146). As such, switching from an anti-PD-1 antibody to an anti-CTLA-4 antibody can functionally be similar to combination therapy using these agents, which may also raise the risk of recurrence of irAEs. Therefore, in the choice of a rechallenge strategy, clinicians need to have a comprehensive assessment of several considerations, such as the patient’s initial response to treatment, discerned resistance mechanisms, the tumor microenvironmental properties, the overall condition of the patient, and the judicious choice of biomarkers. This is a holistic approach that is critical to making precise treatment decisions that optimize patient outcomes while minimizing risks.

## 5 Biomarkers

The broad implementation of ICIs has radically changed outcomes of patients suffering from advanced solid tumors. Nevertheless, understanding which subgroups of patients are most likely to benefit from ICI rechallenge is a formidable and largely unmet clinical challenge (147). While PD-L1 expression is a well-established predictive biomarker for first-line ICI therapy, its predictive value in the more complex scenario of rechallenge has been less apparent (147). This section is devoted to discuss the state of the evidence, predictive mechanisms behind, and limitations inherent in key biomarkers that are important for optimal guidance of ICI rechallenge decisions. We in particular pay attention to PD-L1 expression, dynamic circulating tumor DNA (ctDNA) monitoring, and the neutrophil-to-lymphocyte ratio (NLR). A comprehensive summary of the assay method, predictive values and key considerations for such potential biomarkers is presented in **Table 3**.

**Table 3:** Predictive value of biomarkers in ICI rechallenge.

Biomarker	Assay & Predictive Cut-off	Predictive Value Summary	Notes	Representative Study
PD-L1 Expression	Immunohistochemistry (IHC) • Positive (TPS/CPS ≥1%)	Baseline high expression may correlate with better outcomes, but predictive value in rechallenge remains controversial. Expert consensus favors continuing immunotherapy in PD-L1 high expressors.	Well-established in 1L; affected by tumor heterogeneity and prior therapy in rechallenge.	(44, 63, 87, 98, 99, 110)
ctDNA	Next-generation sequencing (NGS) • Dynamic monitoring; clearance (e.g., decline ≥50%)	Superior for dynamic monitoring. Clearance is associated with significantly superior PFS and OS.	Promising liquid biopsy; reflects real-time tumor burden and clonal evolution. Requires serial monitoring.	(38, 46, 64, 83, 112, 148)
NLR	Peripheral blood calculation • Cut-off: Varies across studies (e.g., <3.8 vs ≥3.8)	Elevated baseline NLR is a negative predictor, associated with shorter PFS and OS.	Easily accessible & inexpensive; but confounded by non-malignant conditions (e.g., infection). Optimal threshold not standardized.	(57, 59, 126)

### 5.1 PD-L1 expression

PD-L1 expression levels have been created and used as a traditional, and widely validated, biomarker for predicting the efficacy of first-line ICI therapy. In the more complex clinical setting of ICI rechallenge, the possible predictive role of PD-L1 expression still receives a great deal of attention. Multiple studies have shown consistent positive correlation between baseline PD-L1 positivity and clinical benefits in ICI rechallenge (63, 98, 100, 149, 150). For example, a retrospective study by Fu et al., in which 176 patients with advanced NSCLC were enrolled, showed that in the subgroup of patients who had futile ICI rechallenge since the disease progression, PD-L1 positivity was significantly associated with an increased PFS in the later treatment schedule (HR = 0.672, 95% CI: 0.477 - 0.947, P = 0.023) (63). This finding therefore suggests that even after progression, tumors with PD-L1 positivity, in which there is an intrinsic immunogenicity or a tumor microenvironment in which the immune system is activated, may be more susceptible to activation of the immune system during rechallenge, thereby facilitating the development of anti-tumor immune responses. Similarly, Geng et al. found significantly longer median PFS in PD-L1-positive patients (6.3 months vs. 3 months, p=0.002) in a clinical trial of 23 patients with metastatic NSCLC (111). Furthermore, the real-world data from the study conducted by Meng et al. of 49 patients with advanced squamous cell carcinoma of the esophagus supported these findings with an increased ORR of 28.6% compared to 8.8% for patients with a baseline PD-L1 Combined Positive Score > or equal to 1 (103). Taken together, these conclusions highlight the fact that baseline PD-L1 expression can be used as a marker of a patient’s potential responsiveness to rechallenge therapy, and that this information could be important for clinical decision-making.

However, the predictive value of PD-L1 expression is not reproducible in all scenarios of ICI rechallenge, particularly when there may be a change of the tumor microenvironment following first treatment. A retrospective study from Tian et al. on 204 patients with advanced NSCLC revealed that PD-L1 expression at the time of initial diagnosis may no longer be an independent predictor of ICI rechallenge efficacy with progression following a round of ICI therapy. Although univariate analysis showed the association between high PD-L1 expression and PFS (HR: 0.61, P = 0.013), this correlation did not still show a statistical significance after multivariate adjustment (87). In a similar vein, a subgroup analysis on 111 patients performed by Feng et al., for whom the PD-L1 status was known only in 47 (42.3%) showed no observed difference in PFS in the rechallenge phase, regardless of the PD-L1 expression levels (<1%, 1-49% or≥50%) (110). Additionally, Crespi et al. found no significant difference in baseline PD-L1 expression between subsequent treatment subgroups (1-49% vs 50-100%) in patients with NSCLC that failed durvalumab consolidation therapy (p = 0.67) (99). These inconsistencies point to the predictive value of PD-L1 expression as context-dependent and determined by factors such as tumor type, history of treatment and the time of disease progression (87). Despite these limitations, the value in the PD-L1 expression in ICI rechallenge cannot be denied. For patients with high baseline PD-L1 expression, expert consensus and several studies strongly support the strategy of “continuation of therapy” often with combination strategies (like platinum-based chemotherapy) to overcome the resistance mechanisms (44). For the most part, experts have agreed that high PD-L1 expression indicates a chronic dependence of the tumor microenvironment on immune checkpoint pathways. Even if progression occurs, these pathways may remain active thus suggesting that immune checkpoint therapy should not be easily abandoned (44). Therefore, the PD-L1 expression levels are an important reference point to evaluate possible patient benefits in ICI rechallenge. However, it needs to be combined with more dynamic and comprehensive methods of assessment that take into account tumor heterogeneity and the previous treatment history so that more precise patient selection and treatment strategies can be developed.

### 5.2 Circulating tumor DNA

In the context of ICI rechallenge, the dynamic monitoring of the ctDNA is becoming recognized as a novel and powerful way of assessing treatment efficacy and clinical decisions. As a non-invasive biomarker of tumor-specific genomic alterations, ctDNA provides real-time information of tumor burden, clonal evolution and treatment response, which can provide invaluable information for complex ICI rechallenge strategies. Several studies have shown unequivocally that there is a significant correlation between ctDNA clearance rates and good outcomes in ICI rechallenge. For example, patients who show >50% clearance of ctDNA have a much longer PFS than patients who do not show clearance of ctDNA (5.6 months versus 3.2 months, respectively) (38, 46). In the case of gastric cancer and colorectal cancer, the high clearance of ctDNA after first-line ICI treatment was associated with an ORR of 25% and mOS of 12 months in the rechallenge setting (64, 151). This phenomenon implies that high clearance of ctDNA may indicate inherent sensitivity to immunotherapy of the tumor and the successful establishment of an effective anti-tumor memory by the immune system, which creates a propitious “treatment window” to be rechallenged (148).

The usefulness of dynamic ctDNA monitoring is especially high in some types of cancer. For example, in nasopharyngeal carcinoma, dynamic changes in plasma Epstein-Barr virus DNA are thought to be an important biomarker for the evaluation of ICI rechallenge efficacy (104, 112). Studies have shown that patients with complete clearance of Epstein-Barr virus DNA (i.e. 0 copies/mL) during first-line therapy showed significantly longer PFS when they received PD-1 ICI rechallenge in the second-line setting compared to those who received chemotherapy alone (112). This provides further support for the correlation between “hot tumor” phenotype and strong immunotherapy activity suggesting that ctDNA may be predictive of the efficacy of a rechallenge such that the tumor responds to immune therapy (112). In addition to efficacy prediction, ctDNA can also show promise in the selection of discontinuation for treatment. In a study of urothelial cancer, the baseline ctDNA genomic instability score was consistently low in patients with early discontinuation of pembrolizumab who had good prognoses. This finding assists in the identification of patient subsets that may be suitable for a “treatment holiday” thus avoiding overtreatment and minimizing cumulative toxicities (83). In summary, dynamic ctDNA monitoring is a good, non-invasive asset in real-time evaluation of tumor response and disease change in ICI rechallenge.

### 5.3 Neutrophil-to-lymphocyte ratio

The NLR, an easy to access and cheap systemic inflammatory marker, has proven unique prognostic value in the context of ICI rechallenge (59, 126). A high NLR often reflects a more pro-tumor inflammatory microenvironment and a less robust anti-tumor immune response and this was predictive of a suboptimal response to ICI therapy (57). To support this, a retrospective study by Musaelyan et al. of 52 patients with metastatic NSCLC who underwent ICI rechallenge showed that a baseline NLR of≥3.8 was an independent negative prognostic factor for both PFS (HR = 5.89, P = 0.001) and OS (HR = 6.80, P = 0.003) (59). Furthermore, in another study by Musaelyan et al. including 43 patients with advanced stage of NSCLC who responded to initial ICI, but later encountered a disease progression, the multivariate analysis also proved a baseline value of absolute neutrophil to NLR of 3.8 or more to be the independent negative predictor for PFS and OS (126). Specifically, patients with a high NLR (≥3.8) had a 3.26-fold higher risk of progressive disease (HR = 3.26) and 5.17-fold higher risk of death (HR = 5.17) compared with those with a low NLR (< 3.8) (126). Katayama et al. also found a significant correlation between NLR ≥5 and poorer PFS (HR = 2.22, p=0.045) in a retrospective analysis of 35 patients with large cell lung cancer from 6 different centers who underwent ICI rechallenge after initial treatment progression (57). These findings collectively highlight the fact that a lower NLR could indicate a more beneficial immune microenvironment and patients may be able to reap more benefit from ICI rechallenge.

While NLR has benefits in accessibility and cost-effectiveness it cannot be ignored as a non-specific marker for inflammation. Various factors influence its numerical value such as infections, trauma or other inflammatory conditions. Moreover, the optimal predictive threshold for NLR also varies among different studies, contributing to the complexity of clinical application of NLR. Consequently, although NLR is an important factor for patient selection and prognostic evaluation of ICI rechallenge, it should be considered an ancillary indicator. Future research should target the investigation of the combination application of NLR with other biomarkers (like PD-L1 expression, dynamic ctDNA changes and tumor mutational burden (TMB)) for the development of more specific and precise multi-factorial predictive models. Dynamic monitoring of the changes of NLR should also be put forward to optimize the treatment strategies and popularize the standardized and individualized ICI rechallenge practices.

## 6 Safety prediction and management of ICI rechallenge

Compared with conventional chemotherapy and radiotherapy, ICI rechallenge is still a new area, which requires in-depth examination of its tolerability. For those patients whose first course of therapy with an ICI was discontinued because of irAEs, the safety profile of rechallenge is of special importance. Therefore, appropriate recognition of irAE risks and their subsequent management play a key role in any ICI rechallenge strategy.

### 6.1 Immunity-related adverse events risk identification

The overall incidence of irAEs after rechallenge is still high and several studies have reported rates between 40-70% (52, 152, 153). This increased recurrence could be indicative of strong immune reactivation after immune stimulation, which could result in a recurrence of autoimmune responses. A retrospective study by Mizuno et al. of 46 patients who initially experienced severe (≥Grade 3) non-endocrine irAEs followed by a rechallenge found that 14 patients (30.4%) developed ≥Grade 2 irAEs post-rechallenge. Of these, eight patients (17.4%) developed recurrent irAEs in the same organ system, whereas four patients (8.7%) developed novel irAEs, and two patients (4.3%) developed recurrent and novel irAEs (52). This brings out the fact that recurrence of irAE is not limited to the first involved site but may involve new organs, making clinical diagnosis and management much more difficult (52). Similarly, a multicenter retrospective study by Pollack et al. found that of 80 patients with metastatic melanoma who had to discontinue combination CTLA-4/PD-1 inhibitor therapy as a result of irAEs, 40% continued to experience irAEs of any grade when re-treated with single-agent PD-1 therapy. Of these, 18% were recurrences of initial irAEs, and 21% were clinically significant, but “different” irAEs (154). This research further explains the correlation between irAEs types and recurrence risk with colitis having a much lower rate of recurrence than other irAEs. In contrast, hepatitis, pancreatitis, pneumonitis and nephritis have a higher tendency for “PD-1-like” toxicities (154).

Furthermore, Park et al.’s study of 62 patients with rare irAEs (RirAEs, defined as an incidence of <1% with monotherapy or <2% with combination therapy) found a 22.6% recurrence rate for original RirAEs after rechallenge, but a higher proportion (37.1%) developed new immune toxicities different than their prior event (155). Even for skin, the most affected organ, the recurrence rates are quite low with careful management but still around 24.3% might lead to ICI treatment discontinuation (156). A further study of Cortazar et al. evaluating 31 patients with acute kidney injury found that 23% of patients had recurrent acute kidney injury on rechallenge and shorter interval of rechallenge (median, 1.4 months) was linked to a higher risk of recurrence (56). These results collectively may highlight the complexity and potential severity of irAEs recurrence patterns and thus the need for clinicians to carefully consider the history and nature of a patient’s irAEs including type, severity, and the amount of time since the last rechallenge before a rechallenge trial is initiated. Specific recommendations for evaluating the risk of recurrence and feasibility of rechallenge for various organ specific irAEs are outlined in **Table 4** in order to provide additional guidance in this complex decision making process. These recommendations are stratified by initial irAE grade and are based on clinical evidence and expert consensus.

**Table 4:** Risk and management recommendations for ICI rechallenge after specific irAEs.

Organ System	Initial irAE Grade	Recurrence Risk upon Rechallenge	Rechallenge Feasibility Recommendation	References
Colitis	Grade 1-2	Low	Feasible. Can be considered after full symptom resolution and corticosteroid discontinuation.	(53, 66, 157)
	Grade 3-4	Moderate (~20-30%)	Extreme caution. Endoscopic confirmation of healing advised. Consider de-escalation (e.g., combo to mono).	(12, 53, 157)
Pneumonitis	Grade 1-2	Moderate	Caution advised. Requires radiographic resolution. Close monitoring.	(8, 67, 128)
	Grade 3-4	High	Generally contraindicated, especially if steroid-refractory or with residual fibrosis.	(8, 54, 67)
Hepatitis	Grade 1-2	Low	Feasible. Can be considered after normalization of liver function tests.	(51, 74)
	Grade 3-4	Moderate (~20-30%)	Caution. Requires normalized LFTs and specialist evaluation.	(51, 55, 73, 79)
Myocarditis	Grade 3-4	Limited data, but very high risk	Contraindicated. Permanent discontinuation of ICI is advised.	(11, 54)
Endocrinopathy	Any Grade	Generally Low	Feasible, usually not a contraindication. Can be considered after stabilization on hormone replacement. Management typically leads to good tolerance.	(11, 75)
Dermatologic	Grade 1-2	Moderate	Generally feasible. Can be considered after rash resolution and symptom control.	(11, 156)
	Grade 3-4	High	Caution. Requires complete healing; consider prophylaxis or de-escalation.	(11, 156)
Rare irAEs	Any Grade	High risk of de novo irAEs	Requires highly individualized assessment. Rechallenge may trigger different irAEs.	(52, 155)

### 6.2 Strategies for irAEs management

Effective irAEs management strategies are not only focused on early warning and timely identification but focus on individualized, stratified and multidisciplinary approaches to balance clinical benefits against risks. For the early identification and continuous monitoring, clinicians need to closely monitor clinical symptoms and signs in patients particularly those related to previous irAEs. Concurrently, monitoring of inflammatory molecules (e.g., NLR), parameters of organ function, and clearance rates of ctDNA can be used as early warning indicators for the onset of irAEs and immunological imbalance (126). Regular imaging examination such as chest CT can help in the early diagnosis of recurrent irAEs, e.g., pneumonitis (128), especially the new ground-glass opacities and infiltrates. Regarding strict criteria for treatment interruption and re-initiation, it is imperative to wait until irAEs symptoms and laboratory parameters are fully resolved or at least decreased to Grade 1 according to CTCAE criteria before even considering rechallenge (53). Furthermore, corticosteroid tapering to physiological doses (prednisone ≤10–20 mg/day equivalent) or complete withdrawal is important so that irAEs recurrence is not masked by corticosteroid effects (52, 53). For high-risk irAEs such as myocarditis and severe neurological toxicities, permanent discontinuation of ICI therapy is recommended and rechallenge should not be considered (54).

Individualized risk stratification and choice of treatment are of paramount importance. Strict selection of patients is the key to reducing the hazard of rechallenge. Mizuno et al. indicated that physicians tend to select patients with irAEs that are relatively manageable and which have a less risk of retreatment (52). For rechallenge after resolution of high-grade irAEs, the choice of less toxic regimens (e.g., de-escalation from combination therapy to single agent PD-1 therapy) is critical (55). Li et al.’s search demonstrated that among patients experiencing high-grade hepatitis and later rechallenged with single agent PD-1/PD-L1, 13.7% treatment discontinuation was due to irAEs. In comparison, this rate was 100% for those administered high-risk ipilimumab rechallenge (55). Moreover, studies have clearly stated that for patients who experienced severe irAEs, re-administering the same ICIs that caused the initial toxicity should be avoided in order to reduce the risk factors of recurrent toxicity (55). That is why careful selection after carefully weighing benefits and risks is essential. For patients with a history of severe irAEs, it is recommended that such drugs, which could cause similar toxicities, are avoided. In addition, multidisciplinary team (MDT) collaboration is important in decision-making in ICI rechallenge. Decisions should always be made based on discussions with the MDT taking full account of the patient’s cancer status, other treatment options, previous severity of irAEs, resolution of toxicity and patient preferences (54). This collaborative model is what ensures a comprehensive evaluation of complex cases and formulation of optimal individualized treatment and risk management plans. Furthermore, research by Badran et al. demonstrates that for patients who have had an episode of immune-related enterocolitis (irEC) and whose symptoms have resolved, concurrent selective immunosuppressive therapy during ICI rechallenge is a good strategy for managing recurrence (157). This study found that selective immunosuppressive therapy concurrent with ICI rechallenge reduced the risk of severe irEC recurrence significantly (OR = 0.34, 95% CI 0.13 to 0.92; p=0.034) (157). This suggests that in certain clinical settings, there is a possibility of using immunosuppressant agents to control and decrease the risk of recurrence in existing irAEs (e.g., irEC), without having any negative effect on overall survival.

## 7 Controversies and challenges

Despite the great improvements made in ICI rechallenge, there are several important controversies and challenges, especially in certain patient populations and in resistance mechanisms. This section discusses in depth two such complex areas: the dilemmas and opportunities of rechallenge in patients with primary refractory disease and the unique difficulties encountered when considering the possibility of ICI rechallenge in elderly people. Understanding these more nuanced aspects is extremely important for the improvement of patient selection and the formation of more personalized and effective treatment strategies.

### 7.1 Rechallenging in primary refractory patients: dilemmas and opportunities

The value of reattempt of ICI therapy (rechallenge) in patients with initial primary resistance (i.e., disease progression with PFS_1_ of less than 6 months) continues to be a major controversy in the field of cancer immunotherapy. These tumors are often marked by certain biological shortcomings, such as low tumor mutational burden, defects in antigen presentation, and a highly immunosuppressive tumor microenvironment (e.g. high density of regulatory T cells or M2 macrophages). These factors are the combined reason for the failure of initial ICI treatment and severely reduce the effectiveness of rechallenge (37, 52, 65). Against this background, several retrospective studies have consistently shown that the efficacy of rechallenge for primary refractory patients is significantly lower than in patients with acquired resistance or toxicity-related discontinuation of treatment. This disparity represents a significant problem for current clinical practice (13, 33, 50, 87, 137, 158, 159). For example, a meta-analysis by Xu et al., that included 442 patients with NSCLC, showed that for patients with initial PD the ORR for rechallenge was only 11.4%, considerably less than that of patients who stopped treatment for other reasons (46.2%) (13). Torasawa et al. further supported this trend among 64 patients with stage III or IV non-small cell lung cancer with an ORR of only 6.7% and mPFS of 2.2 months in the PD group and no patients achieving long-term remission (generally defined as mPFS≥2 years) (37). This is in marked contrast to the results for the non-PD group (ORR 29.4%, mPFS 4.1 months) (37). Similar limitations have been seen in thymic carcinoma patients; the analysis of Shao et al., of 35 patients, found that in 18 patients who progressed on initial therapy and who underwent ICI rechallenge, only 3 patients experienced a partial response, with a low ORR of 16.7% and with an mPFS of only 3.53 months, significantly shorter than the 6.00 months found in the chemotherapy group (n=17) (P = 0.041) (39). Liu et al.’s study of 175 patients with NSCLC who progressed on immuno-chemotherapy and 82 patients (46.86%) who were classified as primary refractory further highlighted this problem (38). The efficacy rate in these patients was much lower compared to those with acquired resistance (ORR: 12.20% vs 20.43%, p = 0.043; PFS: 3.2 months vs 4.5 months, p = 0.012), and the resistance pattern (p=0.007, HR = 0.607) was found to be an independent risk factor for shorter rechallenge PFS in multivariate analysis (38). Additionally, a multicenter retrospective study from Japan dividing 45 patients with unresectable hepatocellular carcinoma who received atezolizumab plus bevacizumab as first-line immunotherapy found that in 25 that received durvalumab plus tremelimumab rechallenge, 3 with an initial best response of PD (n=3) consistently showed PD upon rechallenge, with a 100% concordance rate (3/3) (15). This raises the possibility that for those patients whose initial treatment with an ICI combination therapy failed and resulted in disease progression, an attempt at rechallenge with another combination regimen of ICI’s may have little potential for control of tumor growth. These data strongly suggest that primary refractory tumors may have developed more comprehensive immune evasion mechanisms and it may be difficult to effectively overcome deep-seated barriers to treatment with mere re-administration of same or similar ICI drugs.

Nevertheless, the latest research has demonstrated some positive signals and possible breakthroughs, indicating that primary resistance is not absolutely irreversible (124, 136, 160). Studies on advanced HCC patients have shown that even of those who underwent initial failure of ICI treatment (n=21), 11 patients have experienced partial response or stable disease when switching to different ICI regimens or subsequent treatments (124). Similarly, objective responses were seen in 2 initially refractory HCC patients by Chen et al. in a study of 54 advanced HCC patients after switching ICI drugs for rechallenge (161). Ravi et al. also found three patients (21%) with metastatic renal cell carcinoma who progressed on initial therapy with ICI with objective response after rechallenge (106). Takahara et al. and Scheiner et al. came to similar conclusions (124, 136). These findings together indicate that primary refractory tumors may show heterogeneity with underlying mechanisms that may include dynamic changes in tumor microenvironment, the complementary nature of different ICI mechanisms, and the effect of intermediate treatment steps on the immune status of the tumor (124, 133, 136, 143). This gives new hope to patients with primary refractory disease and suggests the importance of deep understanding of resistance mechanisms, optimal patient selection and innovative treatment strategies for ICI rechallenge.

### 7.2 Unique challenges of ICI rechallenge in elderly patients

Elderly patients are confronted with special and complex challenges with respect to ICI rechallenge, which can be attributed mainly to the reduced tolerance to irAEs, different prognoses and reduced physiological reserves. Matsukane et al’s study of 436 patients with NSCLC (including 104 elderly patients aged ≥75 years) found that although there were no significant differences in the incidence and severity of irAEs between elderly and younger patients, elderly patients were significantly more likely to switch to BSC after irAEs (22.4% vs 11.3%, p=0.026) (162). This result raised the question that elderly patients may have a lower tolerance for the irAEs and are more likely to stop treatment in favor of supportive care. This tendency could potentially reduce the benefits for long-term survival that they obtain from rechallenge. Furthermore, the study showed that younger patients who suffered irAEs had longer OS (HR 0.59, p=0.013), whereas elderly patients did not (HR 0.80, p=0.588) (162). This result suggests that the occurrence of irAEs may not have the same favorable prognosis in elderly patients as in younger patients. The inherent causes could be that elderly patients have more comorbidities, lower organ function reserves, and a relatively weak immune system that makes it difficult for them to fully recover and benefit from further treatment after experiencing irAEs despite control (162). Therefore, in the elderly population, clinical decisions should take into full consideration alternative indicators other than age, such as frailty, sarcopenia, and cachexia, to identify patients who could tolerate irAEs and benefit from them, and the individualized rechallenge strategy should be formulated.

## 8 Discussion and future perspective

This comprehensive review presents the most recent developments in ICI rechallenge in advanced solid tumors, including the selection of patients, therapeutic strategies and efficacy/safety management. Our analysis highlights the fact that patients with a long-lasting first response (PFS_1_ > 6 months), long treatment-free interval (TFI > 6 months), and complete resolution of previous irAEs (to grade ≤1) are the best candidates for rechallenge. Furthermore, combination therapies, for example by combining chemotherapy, anti-angiogenic agents or local radiotherapy, have been found to greatly improve rechallenge efficacy through multi-target mechanisms by modulating the tumor microenvironment.

Despite such major progress, there are currently several significant challenges and limitations in the field of ICI rechallenge. Firstly, most available research is based upon retrospective analyses which are highly prone to selection bias and are often very heterogeneous in terms of pooled results. There is still a large lack of prospective, large scale, high quality clinical trials and this therefore limits the level of evidence to support the conclusions drawn. Secondly, current clinical practice does not have standardized criteria for patient’s selection and best timing for the re-Challenge. While some biomarkers, such as PD-L1 expression, dynamic monitoring of ctDNA and the NLR have a potential predictive value, their stability and universal applicability remain a topic of debate. Moreover, there appears to be a lack of validated multi-factor predictive models to incorporate these different elements to enable precise prediction of efficacy and safety.

Additionally, in certain patient populations, especially the elderly, their reduced tolerance to irAEs and the inability of irAEs to be associated with favorable prognoses as observed in younger patients make a more holistic assessment for beneficiary selection among these groups necessary. The value of rechallenge in different cancer types and in primary refractory patients (e.g., PFS_1_ < 6 months) remains controversial as well and requires further clarification of the underlying that is, the immune mechanisms of mune suppression. Lastly, this review mainly focused on efficacy and safety, yet the long-term economic benefits of ICI rechallenge and comprehensive impact on patients quality of life have not been well studied, which is also another critical consideration in clinical decision-making.

To increase ICI rechallenge from an empirical approach to a precision medicine paradigm, future efforts should focus on addressing the following key challenges: Firstly, there is an urgent need to develop more sophisticated dynamic biomarkers and clinical predictive models. This includes combining multi-dimensional data to build models to identify those patients most likely to benefit, one that predicts how well the treatment may work and potential safety issues so that the treatment can be tailored to the patient. Secondly, it is important to explore and validate novel combination strategies intensively. This research should be focused on the development and evaluation of more effective and safer therapeutic regimens based on different resistance mechanisms in order to achieve the greatest efficacy with less toxicity. Thirdly, the formation of a personalized “monitor-intervene-rechallenge” benevolent cycle is very important. This entails defining the optimal timing and strategies for rechallenge with precision in order to ensure that the patients will achieve maximal clinical benefit with minimal risk. Finally, efforts to make customized research for special populations stronger is imperative. This includes conducting targeted clinical trials for the elderly patients and for patients with primary resistance, in order to optimize their treatment protocol. By addressing these challenges, ICI rechallenge will move from an experience-driven paradigm to one that is based on strong evidence and precision to ultimately provide more effective, safer, and highly personalized treatment options for advanced solid tumors to secure its position in routine clinical practice.

## Data Availability

The original contributions presented in the study are included in the article/supplementary material. Further inquiries can be directed to the corresponding author.
